# Women’s higher likelihood of disability pension: the role of health, family and work. A 5–7 years follow-up of the Hordaland Health Study

**DOI:** 10.1186/1471-2458-12-720

**Published:** 2012-08-31

**Authors:** Inger Haukenes, Sturla Gjesdal, Guri Rortveit, Trond Riise, John Gunnar Mæland

**Affiliations:** 1 Department of Public Health and Primary Health Care, University of Bergen, Kalfarveien 31, NO-5018, Bergen, Norway; 2 Research Unit for General Practice, Uni Health, Bergen, Kalfarveien 31, NO-5018, Bergen, Norway

**Keywords:** Disability pension, Cohort study, Educational status, Gender, Health, Occupational group, Risk factors

## Abstract

**Background:**

Women’s higher risk of disability pension compared with men is found in countries with high female work participation and universal welfare schemes. The aim of the study was to examine the extent to which self-perceived health, family situation and work factors explain women’s higher risk of disability pension. We also explored how these factors influenced the gender difference across educational strata.

**Methods:**

The population-based Hordaland Health Study (HUSK) was conducted in 1997–99 and included inhabitants born in 1953–57 in Hordaland County, Norway. The current study included 5,959 men and 6,306 women in paid work with valid information on education and self-perceived health. Follow-up data on disability pension, for a period of 5–7 years, was obtained by linking the health survey to a national registry of disability pension. Cox regression analyses were employed.

**Results:**

During the follow-up period 99 (1.7%) men and 230 (3.6%) women were awarded disability pension, giving a twofold risk of disability pension for women compared with men. Except for a moderate impact of self-perceived health, adjustment for family situation and work factors did not influence the gender difference in risk. Repeating the analyses in strata of education, the gender difference in risk of disability pension among the highly educated was fully explained by self-perceived health and work factors. In the lower strata of education there remained a substantial unexplained gender difference in risk.

**Conclusions:**

In a Norwegian cohort of middle-aged men and women, self-perceived health, family situation and work factors could not explain women’s higher likelihood of disability pension. However, analyses stratified by educational level indicate that mechanisms behind the gender gap in disability pension differ by educational levels. Recognizing the heterogeneity within gender may contribute to a deeper understanding of women’s higher risk of disability pension.

## Background

Women’s higher risk of sickness absence and disability pension is a consistent finding in countries with universal welfare schemes and high female work participation [1-3]. Different explanations have been suggested, such as women’s poorer self-perceived health, higher burden of musculoskeletal pain, higher prevalence of common mental disorders, greater involvement in family and domestic work, higher preference of part-time positions, and lower status in the labour market [2,4-8].

Self- perceived health is strongly related to disability pension [6], but gender difference in this relation is scarcely examined. A Norwegian population-based study found limited impact of self-perceived health on women’s excess risk of disability pension [1], while moderate impact was found among Helsinki municipal employees regarding medically confirmed sickness absence [4]. With respect to family situation, there is some evidence that financial strain, single parenthood and having a disabled spouse enhance women’s risk of sickness absence [7,9]. Considering disability pension, neither marital status, number of children or age of children seems to explain women’s excess risk [4,10]. However, conflicting results and a lack of studies using disability pension as outcome make it difficult to conclude.

Higher levels of education is associated step by step with increased health and lower risk of disability pension [11-13]. However, the recent gender equalization in educational achievement in Norway has not reduced the gender gap in disability pension. One reason may be that equality in educational achievement does not necessarily imply gender equality in occupational status. Women are more often employed than men in the public sector, work part time and occupy lower working-class positions with lower incomes, and are thus more exposed to work factors associated with disability pension [5,8,14]. Considering the steep educational gradient in risk of disability pension, most likely risk-factors and their impact differ across strata of education. Also, a Norwegian study found that it is far more challenging to explain disability risk among the lower educated than among the higher educated [14]. These findings point at educational levels as relevant strata for examining gender differences in disability pension. However, this perspective has not been pursued in literature.

The aim of the study was to examine the extent to which self-perceived health, family situation and work factors explain women’s higher risk of disability pension. We also explored how these factors influenced the gender difference across educational strata.

## Methods

### Study population

The population based Hordaland Health Study (HUSK) was conducted in 1997–99 and included inhabitants born in 1953–57 in Hordaland County, Norway. HUSK was a collaboration between the National Health Screening Service, the University of Bergen and local health services. A total of 8,598 men and 9,983 women (age 40–45) participated, yielding a participation rate of 57% for men and 70% for women. Data collection was performed in two steps. Firstly, all participants underwent a physical health examination and completed a self-administered questionnaire. In the second step the participants were randomized in four equal groups (two male and two female groups). Each of these groups was given a questionnaire with gender-specific questions and general questions about family and work and a total of 7,327 men and 8,843 women answered. The response rate in the second step was 85,4% for the men and 87,1% for the women (% of participants). The sub-sample used in the current study was based on the second questionnaire and included participants who reported being in paid work (never awarded disability pension before participating in HUSK) and with valid information on educational level and self-perceived health. These criteria led to an exclusion of 1090 men and 2447 women. Further, farmers were excluded (268 men and 72 women) due to their special working conditions compared with the rest of the work force. Finally, individuals awarded disability pension the first 12 months after participating in HUSK were excluded (10 men and 18 women), in order to eliminate report bias as a result of already being in the process of applying for a disability pension [15]. The final study population consisted of 5,959 men and 6,306 women.

### Outcome

Follow-up data on disability pension was obtained by linking the health survey to a national register of disability pension by means of the unique personal ID number. The outcome was award of at least 50% disability pension during follow-up, from 12 months after participating in the heath survey (HUSK) until the end of 2004 (5–7 years follow-up period). For all disability pensioners, the time interval between the date of participation in HUSK and the date of the disability pension award was calculated.

### Self-perceived health

Self-perceived physical and mental health status was measured by the self-report Short Form−12 (SF-12), a validated questionnaire with well-documented psychometric properties [16]. This shorter version of the SF-36 is recommended for large population surveys such as HUSK. The questions in SF-12 mainly assess the individuals’ perceived health-related limitations in daily activity, work and social relationships, thus giving a general indication of self-assessed functional ability. Weighted summary scores for perceived mental and physical health were standardized in accordance with the US norm data with a mean score of 50 (SD 10) [16]. In the analyses, the scores were divided into quartiles with the lowest quartile implying the poorest self-perceived health [17].

### Family situation

Information on marital status and children in the household were used to generate three dichotomous variables: married (yes/no), ever divorced (yes/no), children < 18 years in the household (yes/no). Another dichotomous variable signifying whether or not the spouse received social security benefits, was generated from information on the spouse’s status regarding sickness/rehabilitation allowance, disability pension and unemployment benefits.

### Working hours per week

The participants reported number of paid working hours per week. We recoded the information into three main categories: full-time (≥37 hours), part-time (27–36 hours), and small part-time (<27 hours).

### Occupational class

Self-reported information on branch of industry and occupation was manually converted into four-digit codes based on the International Standard Classification of Occupations, ISCO-88 (COM) [18]. Using an internationally applicable algorithm by Ganzeboom and Treiman [19], ISCO codes were recorded into the Eriksson, Goldthorpe and Portocareros occupational class scheme [20]. The following occupational classes were used: (1) Administrative and professional, (2) routine non-manual, and (3) manual. Among the first class are managers, directors, senior officials and academic professions that require at least 4 years of education. Examples of occupations in the second class are nurses, social workers, teachers in compulsory and vocational schools, clerks and home-helpers. The category of manual workers includes skilled and unskilled workers within crafts, industry, construction and service. The occupational class scheme has achieved a high degree of comparability between European countries when measuring morbidity [21]. The scheme also reflects an occupational structure of status that is associated with disability pension for both genders [14,22].

### Education

Information on education was acquired through responses to the following question: “What is the highest level of education you have achieved?” Options for answers were: University ≥ 4 years, college < 4 years, higher secondary school, vocational school, and primary + lower secondary school. We applied the following three educational levels: University/college; vocational/upper secondary and primary/lower secondary.

### Analyses

We employed chi-square tests to examine the gender difference in distribution of participants by strata of independent variables. Univariate Cox regression analyses were employed to examine the association between each independent variable and disability pension, stratified by gender. To examine the gender difference in risk of disability pension, we employed a multivariate Cox regression analysis. To test whether the relationship between the covariates and risk of disability pension was different between men and women (effect modification) we included interaction terms in the Cox model when analysing the total cohort.

The independent variables were added in a predefined order, introducing self-perceived mental and physical health first: second, variables concerning family situation, followed by occupational class and, finally, working hours per week. The impact of additional health measures (self-reported medical conditions, somatic symptoms and mental health assessed by the Hospital Anxiety and Depression Scale) were also tested in the model.

The results are presented as hazard ratios (HR) with 95% confidence intervals (CIs). The change in gender HR when introducing covariates in the model was estimated in percent by the following formula: (HR _adjusted_ – HR _unadjusted_)/HR _unadjusted_ *100. A Spearman's Rank Order correlation was run to determine the relationship between educational levels and occupational class. All covariates were tested for the assumption of proportionality. No marked deviation from the proportional hazard assumption was found. The analyses were performed using SPSS (PASW) 18.0 for Windows.

### Ethical approval

The study protocol was approved by the Regional Committee for Medical Research Ethics, Western Norway and by the Norwegian Data Inspectorate. Written statements of informed consent were gathered from all the participants in the current study at the time of the physical health examination.

## Results

### Gender difference across independent variables

The difference in the distribution of men and women across all independent variables was statistically significant, except for marriage (yes/no) and living with children (yes/no) (Table 1). There were marked differences in the distribution of men and women by occupational class and working hours per week (Table 1). A relatively higher proportion of men reported administrative and professional work, while routine non-manual work was relatively more often reported by women. Furthermore, 51% of the women had part-time work compared to 11% of the men.

**Table 1:** Distribution of participants, cumulative incidence of disability pension (DP) and unadjusted risk of DP by covariates

	**Distribution**					**Disability pension**							
	**Men**		**Women**			**Men**				**Women**			
	**n**	**%**	**n**	**%**	p-value*	**n**	**%**	**HR**	**95% CI**	**n**	**%**	**HR**	**95% CI**
Gender	5959		6306			99	1.7			230	3.6		
Married					0.269								
Yes	4481	75.2	4796	76.1		62	1.4	1.00		169	3.5	1.00	
No	1478	24.8	1510	23.9		37	2.5	1.85	1.23–2.78	61	4.0	1.18	0.88–1.59
Ever divorced					0.000								
No	5487	92.1	5619	89.1		83	1.5	1.00		196	3.5	1.00	
Yes	472	7.9	687	10.9		16	3.4	2.31	1.35–3.94	34	4.9	1.47	1.02–2.11
Living with children < 18 years					0.206								
Yes	4508	75.7	4604	73.0		66	1.5	1.00		146	3.2	1.00	
No	1068	17.9	1158	18.4		22	2.1	1.43	0.88–2.32	58	5.0	1.63	1.20–2.21
Spouse receive social benefit					0.000								
No	5412	90.8	5940	94.2		84	1.6	1.00		210	3.5	1.00	
Yes	547	9.2	366	5.8		15	2.7	1.77	1.02–3.06	20	5.5	1.54	0.98–2.44
Educational level					0.000								
University/college	2402	40.3	2353	37.3		26	1.1	1.00		48	2.0	1.00	
Vocational/higher secondary	2750	46.1	2874	45.6		47	1.7	1.55	0.96–2.50	106	3.7	1.80	1.28–2.53
Primary/lower secondary	807	13.5	1079	17.1		26	3.2	2.94	1.71–5.06	76	7.0	3.47	2.41–4.98
Occupational class					0.000								
Administrative/professional	2908	48.8	1481	23.5		33	1.1	1.00		27	1.8	1.00	
Routine non-manual	906	15.2	3128	49.6		10	1.1	0.97	0.48–1.97	102	3.3	1.77	1.16–2.71
Manual	2010	33.7	1555	24.7		52	2.6	2.25	1.46–3.49	94	6.0	3.30	2.15–5.06
Working hours per week					0.000								
≥37 hours	5174	86.8	2888	45.8		77	1.5	1.00		90	3.1	1.00	
27-36 hours	549	9.2	1769	28.1		11	2.0	1.33	0.71–2.50	55	3.1	0.98	0.70–1.37
<27 hours	102	1.7	1463	23.2		5	4.9	3.38	1.37–8.37	78	5.3	1.69	1.25–2.29
Self-perceived mental health					0.000								
Quartile 1 (good)	1626	27.3	1338	21.2		14	0.9	1.00		54	4.0	1.00	
Quartile 2	1640	27.5	1636	25.9		14	0.9	0.99	0.47–2.09	47	2.9	0.71	0.48–1.04
Quartile 3	1351	22.7	1501	23.8		20	1.5	1.73	0.87–3.42	35	2.3	0.57	0.37–0.88
Quartile 4 (poorest)	1342	22.5	1831	29.0		51	3.8	4.50	2.49–8.14	94	5.1	1.28	0.91–1.79
Self-perceived physical health					0.000								
Quartile 1 (good)	1530	25.7	1641	26.0		6	0.4	1.00		18	1.1	1.00	
Quartile 2	1483	24.9	1478	23.4		7	0.5	1.20	0.40–3.56	21	1.4	1.28	0.68–2.40
Quartile 3	1669	28.0	1410	22.4		20	1.2	3.03	1.22–7.55	30	2.1	1.92	1.07–3.44
Quartile 4 (poorest)	1277	21.4	1777	28.2		66	5.2	13.37	5.80–30.84	161	9.1	8.59	5.28–13.99

*χ^2^ for gender differences in distribution of participants.

Hazard ratios (HR) and 95% confidence intervals (95% CI) from univariate Cox regression analyses.

### Gender difference in disability pension

During the 5–7 years follow-up period, a total of 99 (1.7%) men and 230 (3.6%) women were awarded disability pension (Table 1). In both genders, lower education, manual work, part-time work and lower self-perceived physical health were associated with disability pension.

Regarding self-perceived mental health, men with the lowest scores (quartile 4) had a substantially higher risk of disability pension compared with the reference group (quartile 1). Among women the pattern was unclear, but the reference group had a significantly higher risk of disability pension compared with those in quartile 3. This difference in effect between men and women was significant (test of interaction in total cohort: p = 0.001). When estimating this effect of interaction, by analysing the effect of gender in strata of self-perceived mental health (data not shown), we found no gender difference in risk of disability pension among those in the lowest quartiles of self-perceived mental health.

For men, being unmarried or having a spouse on social benefits increased the risk of subsequent disability pension, while for women a higher risk was related to not having children in the household. However, none of these differences were significant (test of interaction).

In the multivariate Cox regression analysis women’s higher risk of disability pension (crude HR = 2.21, 95% CI = 1.75–2.80) was attenuated with a 15% reduction in the hazard ratio, when adjusting for self-perceived mental and physical health (Table 2). Additional adjustments for variables pertaining to family situation, occupational class and working hours per week did not further reduce women’s excess risk, leaving a substantial unexplained gender difference in the final model (HR = 1.95, 95% CI = 1.43–2.65). Adjustment for additional health measures did not contribute to further explaining the gender difference, and were not incuded.

**Table 2:** Risk of disability pension among women compared to men, in a cumulative Cox regression model

	**Model 1**		**Model 2**		**Model 3**		**Model 4**		**Model 5**	
	**HR**	**95% CI**	**HR**	**95% CI**	**HR**	**95% CI**	**HR**	**95% CI**	**HR**	**95% CI**
Men	1		1		1		1		1	
Women	2.21	1.75–2.80	1.87	1.48–2.37	1.91	1.48–2.46	2.04	1.56–2.68	1.95	1.43–2.65
Mental health (ref = Quartile 1)			1		1		1		1	
Quartile 2			1.17	0.82–1.65	1.20	0.83–1.75	1.23	0.84–1.78	1.24	0.85–1.83
Quartile 3			1.02	0.71–1.46	1.10	0.75–1.59	1.10	0.75–1.62	1.12	0.76–1.65
Quartile 4			2.04	1.53–2.72	1.98	1.45–2.71	1.96	1.43–2.70	1.96	1.42–2.72
Physical health (Quartile 1)			1		1		1		1	
Quartile 2			1.49	0.86–2.59	1.69	0.94–3.03	1.63	0.91–2.93	1.63	0.91–2.92
Quartile 3			2.50	1.53–4.08	2.70	1.60–4.58	2.49	1.47–4.24	2.34	1.37–4.00
Quartile 4			10.32	6.76–15.75	10.59	6.67–16.81	9.19	5.77–14.63	8.64	5.42–13.77
Married (ref = yes)					1		1		1	
No					0.78	0.53–1.14	0.76	0.51–1.13	0.76	0.50–1.14
Ever divorced (ref = no)					1		1		1	
Yes					1.83	1.17–2.86	1.75	1.11–2.77	1.86	1.16–2.97
Children < 18 y at home (ref = yes)					1		1		1	
No					1.51	1.14–1.99	1.47	1.11–1.95	1.56	1.17–2.07
Spouse social benefit (ref = no)					1		1		1	
Yes					1.49	1.03–2.17	1.44	0.99–2.09	1.52	1.05–2.21
Administrative/professional class							1		1	
Routine non-manual class							1.22	0.87–1.73	1.23	0.86–1.74
Manual class							2.09	1.52–2.87	2.12	1.51–2.94
>37 working hours per week									1	
27-36									0.88	0.63–1.23
<27									1.46	1.05–2.02

Hazard ratios (HR) and 95% confidence intervals (95% CI) from multivariate Cox regression models.

Model 1: Gender only.

Model 2: Model 1 + self-perceived health (mental and physical).

Model 3: Model 2 + family situation (married/divorced, living with children <18 years, spouse receives social benefit).

Model 4: Model 3 + occupational class.

Model 5: Model 4 + working hours per week.

Repeating the analyses in strata defined by educational level, self-perceived health, occupational class and working hours per week were clearly associated with a risk of disability pension among highly educated women, while only self-perceived health was associated with lower educated women (Table 3). The disability risk among women with high education (crude HR = 1.88, 95% CI = 1.17–3.03) was fully explained in the final model (HR = 1.10, 95% CI = 0.58–2.08), whereas this was not the case for women with lower education. The correlation (r_s_) between educational level and occupational class was 0.507, *p <* 0.001.

**Table 3:** Risk of disability pension among women compared to men in three educational groups

**Educational level**	**Model 1**		**Model 2**		**Model 3**		**Model 4**		**Model 5**	
	**HR**	**95% CI**	**HR**	**95% CI**	**HR**	**95% CI**	**HR**	**95% CI**	**HR**	**95% CI**
University / college										
Women (men = ref)	1.88	1.17–3.03	1.48	0.91–2.39	1.69	1.00–2.86	1.45	0.82–2.57	1.10	0.58–2.08
Vocational/high secondary										
Women (men = ref)	2.19	1.55–3.08	1.98	1.40–2.80	1.96	1.36–2.81	2.40	1.63–3.54	2.14	1.37–3.37
Primary/secondary										
Women (men = ref)	2.22	1.42–3.47	1.86	1.19–2.91	1.88	1.15–3.05	1.99	1.18–3.35	2.67	1.50–4.77

Hazard ratios (HR) and 95% confidence intervals (95% CI) from multivariate Cox regression models.

Model 1: Gender only.

Model 2: Model 1 + self-perceived health (mental and physical).

Model 3: Model 2 + family situation (married/divorced, living with children <18 years, spouse receives social benefit).

Model 4: Model 3 + occupational class.

Model 5: Model 4 + working hours per week.

## Discussion

### Main results

In a Norwegian cohort of middle-aged men and women, we found no adequate explanation for women’s higher likelihood of disability pension, except for a moderate impact of self-perceived health. Further adjustment for family situation, occupational class and working hours per week did not influence women’s higher disability risk in the total cohort. However, in analyses stratified by educational level, these factors fully explained women’s excess risk of disability pension among the highly educated, but not among the less educated.

### Self-perceived health

Some have argued that health plays only a minor role in exit from working life in modern societies [23]. However, several studies have shown that self-perceived health is a strong predictor of subsequent sickness absence and disability pension across gender and social class [6,17,24-26]. In the current study self-perceived physical health was strongly associated with disability pension in both genders, while self-perceived mental health was associated only among men. However, in the multivariate analyses both dimensions of self-perceived health contributed to explain women’s excess disability risk. This impact was found in the total cohort and in strata of educational level. A Norwegian study found that among individuals on sick leave for more than eight weeks due to musculoskeletal disorders, women less than 50 years of age had a higher risk of disability pension compared with men in the same age group [5]. Self-perceived physical health may reflect musculoskeletal disorders in both genders, but the higher impact on women’s disability risk may be due to an earlier chronicity among women. Further, Laaksonen et al. found that self-perceived health and self-reported diagnoses explained the gender difference in long-term sickness absence among middle-aged [4]. In a Norwegian study, self-perceived global health had little impact on women’s excess risk, while adding mental distress significantly reduced the gender difference [1]. However, the addition of several self-reported health measures in the current study provided no further explanation of women’s excess risk of disability pension.

The relation between self-perceived mental health and risk of disability pension did not display a linear trend, a finding that may be related to the construction of the physical and mental summary SF-12 scales providing uncorrelated (orthogonal) factors, a construct that is theoretically unlikely [27]. Using the multidimensional SF-36 questionnaire, Laaksonen et al. found a strong association between self-perceived physical health and sickness absence while mental health was only weakly associated [26]. Studies using the Hospital Anxiety and Depression Scale (HADS), on the other hand, have found linear associations between higher scores on self-reported anxiety/depression and subsequent disability pension [28,29].

### Family situation

In line with our findings, a Norwegian study found that living with children younger than 7 years of age diminished the likelihood of disability pension among women [13]. However, the current study supports the general impression that marital status, children in the household or a retired spouse all have little impact on the gender difference in disability pension [4,5,8]. Women’s increased participation in working life has brought about some changes in traditional family roles and expanded the use of part-time jobs. With respect to the interface between work and family, findings indicate that both genders are at risk of sickness absence when experiencing negative interference from work on the family situation [30,31].

### Occupational social class

As confirmed in the literature, the risk of disability pension increases substantially with lower occupational social class [14,22]. Some studies have suggested that women’s excess risk of sickness absence and disability pension is more likely explained by vertical gender segregation than by horizontal segregation [2,32]. Vertical segregation refers to the unequal gender distribution in occupational social class, while horizontal segregation denotes the tendency of men and women to work in different occupations [33]. In the current study, controlling for occupational class (vertical segregation) in the total cohort, did not contribute towards explaining women’s excess risk of disability pension, thus confirming the findings from a previous Norwegian study [1].

Nevertheless, in the analyses stratified by educational level we found that the risk of disability pension among highly educated women was considerably influenced by occupational class. One explanation is that higher education among women, more frequently than among men, is translated into occupational classes with less power, less autonomy and less status and thereby implies an excess risk of disability pension [34]. In the current study, the different distribution of men and women by occupational class supports this interpretation. Among the lower educated, occupational class did not explain women’s higher disability risk, thus supporting studies that find male workplaces within the manual occupations particularly hazardous to health [32,35].

### Part-time work

In the current study, part-time work less than 27 hours per week was associated with increased risk of disability pension in both genders, but did not contribute to explain women’s higher risk of disability pension in the total cohort [5,13]. One might question whether the risks associated with part-time work are linked with the reduced number of hours at the work place, and conversely the higher number of hours outside the work place, or are merely a reflection of poor health and reduced work ability among part-time workers. Considering the unequal distribution of men and women in part-time positions, reasons for part-time work most likely differ by gender. In the current study, the relatively high risk of disability pension among men working part-time indicates reduced work ability. Among women, however, part-time work is often preferred to ease the double burden of work and family obligations [7,36]. When working hours per week did not influence the gender difference in disability pension in the total cohort, this may be related to the unequal gender distribution among part-time workers and the selection of men with reduced work ability into part-time positions.

However, in the analyses stratified by education, working hours per week contributed to explain women’s excess risk of disability pension among the highest and second highest educated, but not among the lowest educated. Again, the impact of gendered distribution, selection effects and adjustment for health make it difficult to interpret the findings. In conclusion, working hours per week is an ambiguous variable that needs to be combined with additional information when interpreting impact on gender difference in disability pension.

### The unexplained risk

Population-based studies of risk factors for disability pension give valuable information about risks that are common for both genders and for men and women separately [11,12,14]. However, there is a need for studies that aim at examining the gender difference as such [1]. In the current study, the substantial unexplained disability risk among women versus men, after adjusting for health, family and work factors, mirrors a complexity that may need a gender difference approach in order to be understood.

Firstly, occupational health research has accumulated far more knowledge of health hazards related to male working life than female [37]. Secondly, widely used tools to assess the psychosocial working environment were developed within a male working force paradigm, and may lack essential perspectives related to women’s multiple roles in society [38,39]. Emotional demands, rewards at work, management quality and role conflicts may be especially important for explaining the gender gap in long-term sickness absence and disability pension [34]. Thirdly, education in modern society is not a onetime occurrence, but rather a continuous process throughout the working career. The ability to cope with changing work tasks, technology and decision processes is probably vital for conceiving work as a meaningful and significant part of one’s life. Lower education combined with part-time work gives women fewer options regarding jobs, less opportunity to attend training, less coping experience and a higher risk of exclusion as a result of reorganization and downsizing. These factors need to be addressed in further studies of gender difference.

### Limitations

Firstly, the response rate was higher for women than for men, 70% versus 57% respectively, which opens for possible selection bias related to genders. A study of nonparticipants in HUSK found that 5.5% of the male nonparticipants and 8.8% of the females were awarded disability pension after the HUSK survey was performed. Among participants the percentages were 2.7% for men and 5.2% for women. However, the study found no significant gender differences in the association between nonparticipation and awards of disability pension [40]. This finding indicates that the gender distribution in cumulative incidence of disability recipients in the current study was probably not biased by the gender difference in nonparticipation.

Secondly, typical for non-participants in population-based studies are lower educational level and lower income [41]. Since this under-representation related to level of education and income, is most likely similar for men and women, we do not think that our risk estimates are flawed by selective participation. Further, the relatively low incidence of male disability pensioners may impose some restrictions on the interpretation of the analyses. Finally, the information collected at baseline was cross-sectional, thus preventing identification of causal paths between independent variables.

### Strengths

Our study is based on a linkage between HUSK and the National Insurance Administration’s records of disability pensions awarded to Norwegian inhabitants from 1992 onwards. This register is complete, accurate and independent of exposure data obtained in the Hordaland Health survey. The study thus avoids the problem of attrition. The self-reported information at baseline was collected without participants or administrators being aware of future research hypotheses.

Further, the follow-up period from 1997 till 2004 was a period without major changes in Norwegian disability policy with the potential to alter the cumulative incidence by gender. Only minor fluctuations in incidence of disability pension awards were present during this period. The study design excluded individuals awarded disability pension up to 12 months after baseline, thus decreasing the risk of biased information from participants in the process of being awarded disability pension [42]. Also, the current birth cohorts represent an age group with few incidences of pregnancies and maternity leaves that may influence baseline reporting of health and working hours per week.

## Conclusion

Except for a moderate impact of self-perceived health, family situation and work factors did not explain women’s higher risk of disability pension in a middle-aged cohort. However, in analyses stratified by educational level, these factors explained women’s excess risk of disability pension among the highly educated, but not among the lower educated.

Our results indicate that mechanisms behind the gender gap in disability pension differ by educational levels. Recognizing the heterogeneity within gender may contribute to a deeper understanding of women’s higher risk of disability pension.

## Competing interests

The authors declare that they have no competing interests.

## Authors' contributions

IH conceived the study, performed the data analysis, coordinated the study and drafted the manuscript. SG participated in the interpretation of the results and revised the manuscript for important content. GR participated in interpretation of the results and revised the manuscript for important content. TR advised the statistical analyses and revised the manuscript for important content. JMG participated in conceiving the study, advised the statistical analyses, interpretation of the results and drafting the manuscript. All authors have read and approved the final manuscript.

## Pre-publication history

The pre-publication history for this paper can be accessed here:

http://www.biomedcentral.com/1471-2458/12/720/prepub
